# Class I ADP-Ribosylation Factors Are Involved in Enterovirus 71 Replication

**DOI:** 10.1371/journal.pone.0099768

**Published:** 2014-06-09

**Authors:** Jianmin Wang, Jiang Du, Qi Jin

**Affiliations:** MOH Key Laboratory of Systems Biology of Pathogens, Institute of Pathogen Biology, Chinese Academy of Medical Sciences and Peking Union Medical College, Beijing, People's Republic of China; National Institute of Health - National Cancer Institute, United States of America

## Abstract

Enterovirus 71 is one of the major causative agents of hand, foot, and mouth disease in infants and children. Replication of enterovirus 71 depends on host cellular factors. The viral replication complex is formed in novel, cytoplasmic, vesicular compartments. It has not been elucidated which cellular pathways are hijacked by the virus to create these vesicles. Here, we investigated whether proteins associated with the cellular secretory pathway were involved in enterovirus 71 replication. We used a loss-of-function assay, based on small interfering RNA. We showed that enterovirus 71 RNA replication was dependent on the activity of Class I ADP-ribosylation factors. Simultaneous depletion of ADP-ribosylation factors 1 and 3, but not three others, inhibited viral replication in cells. We also demonstrated with various techniques that the brefeldin-A-sensitive guanidine nucleotide exchange factor, GBF1, was critically important for enterovirus 71 replication. Our results suggested that enterovirus 71 replication depended on GBF1-mediated activation of Class I ADP-ribosylation factors. These results revealed a connection between enterovirus 71 replication and the cellular secretory pathway; this pathway may represent a novel target for antiviral therapies.

## Introduction

Enterovirus 71 (EV71) is a member of the genus *Enterovirus* in the family of *Picornaviridae*. EV71 infections are known to cause hand, foot, and mouth disease. However, some severe cases may present with serious neurological symptoms, such as aseptic meningitis, encephalitis, and acute flaccid paralysis; furthermore, infections may lead to death [1], [2]. EV71 has a ∼7.4 kb, positive-sense, single-stranded RNA genome. The genome comprises a single open reading frame that encodes a polyprotein, flanked by 5′- and 3′-untranslated regions (UTRs). The translated polyprotein is cleaved in both *cis* and *trans* processes that involve virus-encoded proteases, 2A^pro^, 3C^pro^, and 3CD^pro^. The cleavages produce about 10 final products and several cleavage intermediates.

All RNA viruses with a positive-strand genome undergo RNA replication upon association with the membranes of infected cells [3]. However, picornaviruses, including EV71, generally do not use native organelle membranes for replication. Instead, they induce the formation of novel cytoplasmic vesicular compartments in infected cells [4], [5]. Several studies have shown that picornavirus-induced vesicle formation is associated with coat protein complex I (COPI) and COPII-mediated vesicle budding from the endoplasmic reticulum and Golgi apparatus [6], [7], [8]. The enteroviral proteins 2B, 2C, and 3A were also reported to be involved in the enterovirus-directed vesicle formation process [9], [10], [11]. However, the mechanisms and pathways involved in EV71-directed membrane reorganization are poorly understood. The involvement of the cellular secretory pathway was suggested by the fact that the fungal metabolite, brefeldin A (BFA), inhibited enteroviral RNA replication [12], [13].

BFA specifically inhibits the activation of small cellular GTPases, which are members of the ADP-ribosylation factor (Arf) family. To date, six members in the Arf family have been identified, and most (all except Arf2) are expressed in human cells. Based on amino acid sequences, the Arfs were grouped into three classes as follows: Class I (Arfs 1–3), Class II (Arfs 4 and 5), and Class III (Arf6) [14]. These proteins participate in the formation of coated membranous vesicles that originate from different organelles. They are key regulators of the cellular secretory pathway. Arf6 is the most divergent of the Arf proteins; it is generally bound to membranes, and it regulates endocytic traffic and actin at the plasma membrane [15], [16]. In contrast, Arfs 1–5 are soluble proteins that cycle on and off membranes. The GDP-bound form of Arf (Arf-GDP) is inactive and resides in the cytoplasm. When Arf-GDP undergoes a nucleotide exchange by binding GTP (Arf-GTP), it becomes activated and associates with membranes. Arf-GTP is required to interact with different membrane proteins, and it initiates the formation of secretory vesicles. The membrane-bound Arf can recruit a wide variety of cellular factors to membranes [17], which in turn, might facilitate virus replication. It has been reported that the activation of Arf1 and its association with membranes are required for poliovirus replication in vivo and in vitro [18]. Arf1 was also reported to play essential roles in the replication of other RNA viruses, including hepatitis C virus and plant red clover necrotic mosaic virus [19], [20]. Arf4 and Arf5 were found to be required for the efficient secretion of dengue viruses [21].

The generation of Arf-GTP from Arf-GDP requires the activity of guanine nucleotide exchange factors (GEFs). There are three identified human GEFs that are inhibited by brefeldin A (BFA), including GBF1, BIG1, and BIG2. Although the activities of all three GEFs can be blocked by BFA, their functions are not identical. GBF1 and the two BIGs localize to *cis*- and *trans-*compartments, respectively, of the Golgi complex. GBF1can activate both Class I and Class II Arfs in cells [22]. In contrast, the knockdown of BIG1 and BIG2 only caused the association of Arf3 to membranes [23]. Furthermore, GBF1 regulates COPI recruitment to *cis*-Golgi compartments [24]; and BIG1 and BIG2 are mainly associated with trafficking to and from the *trans*-Golgi network [25].

The knockdown of GBF1 was shown to reduce replication of poliovirus and Coxsackievirus B3 in vivo [9], [26].

In this study, we investigated the differential involvement of Arf proteins and GEFs in EV71 replication. We reported that Class I Arfs was upregulated with EV71 infection. Simultaneous depletion of Arf1 and Arf3 substantially inhibited viral replication. GBF1 was also identified as a cellular factor crucial for EV71 replication in cells.

## Results

### Expression of Class I Arfs is upregulated with EV71 infection

**Figure 1:**
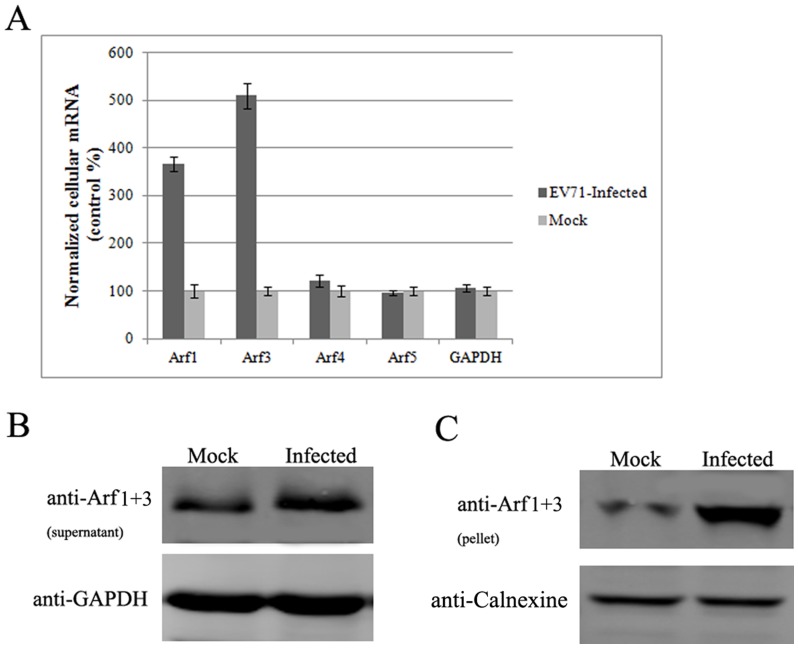
Class I Arfs are upregulated and activated in EV71 infections. (A) Transcripts for both Arf1 and Arf3 are upregulated in EV71-infected cells (*P*<0.05). (B) Expression levels of Class I Arf proteins are upregulated with EV71 infection. (C) Class I Arfs are activated upon EV71 infection.

We previously showed that cellular COPI activity was required for EV71 replication in rhabdomyosarcoma (RD) cells [11]. The recruitment of COPI for the viral coat required an association of Arf proteins with the membrane [27]. Thus, in the present study, we tested the involvement of Arfs in EV71 replication. RD cells were infected at a multiplicity of infection (MOI) of one. After 6 h, the total cellular RNA was extracted, and the copy numbers of four Arf mRNAs (Arf1 and Arfs 3–5) were determined by quantitative real-time PCR. Figure 1A shows that the transcripts for cellular Arf1 and Arf3, but not other Arfs, were noticeably upregulated upon EV71 infection. The expression levels of Class I Arf proteins were also detected by Western blot (Figure 1B). As expected, the expression of Class I Arfs increased noticeably. The membrane-associated protein fraction was collected by centrifugation. Immunoblots probed with anti-Arf1+3 antibodies indicated the activation levels of Class I Arfs. As shown in Figure 1C, the membrane-bound form of Class I Arfs increased substantially with EV71 infection. These results indicated that Class I Arfs were upregulated and activated upon an EV71 infection.

### Knockdown of a single Arf does not affect EV71 replication

**Figure 2:**
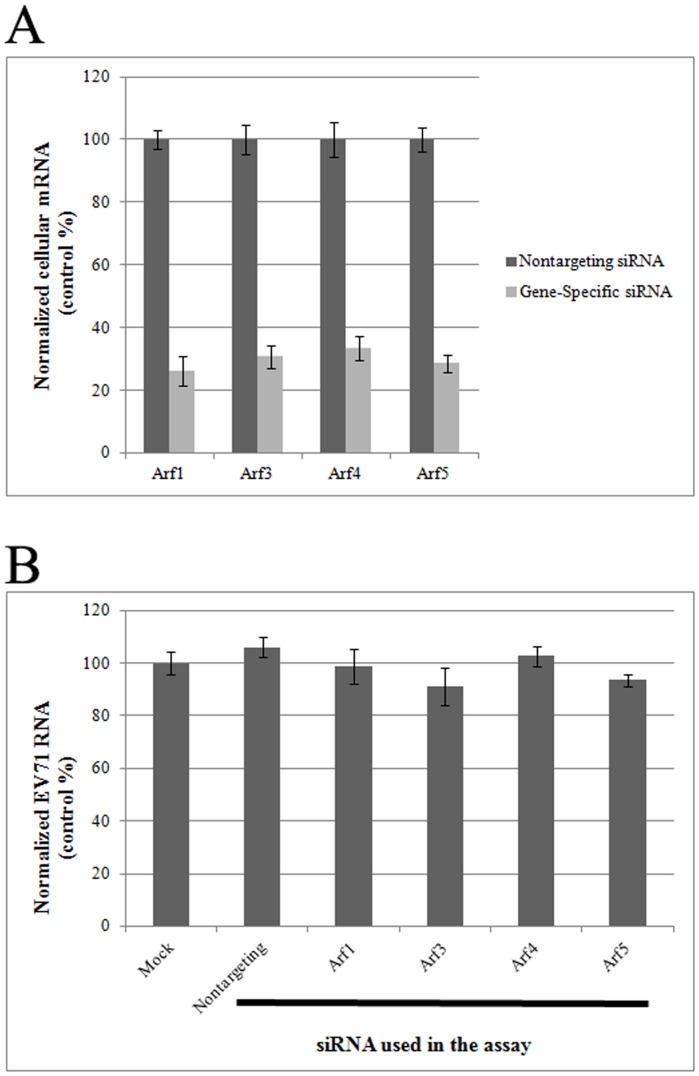
Knockdown of a single Arf does not affect EV71 replication in cells. (A) Individual Arf isoforms were effectively knocked-down with siRNAs (*P*<0.05). (B) Knockdown of a single Arf did not affect EV71 replication in cells (*P*>0.05).

To explore whether the Arf proteins were required for EV71 replication, RD cells were depleted of individual Arfs by transfecting with specific siRNAs. The efficiency of each knockdown was analyzed quantitatively by evaluating the levels of individual Arf mRNA transcripts. The results showed that siRNA treatment reduced the transcript level of each Arf by 65–75% (Figure 2A). To examine the effects of the Arf knockdowns on EV71 RNA accumulation, the knockdown cells were infected with EV71 at 1 MOI. Control siRNA-treated RD cells were also infected. At 6 h post infection (hpi), EV71 replication was evaluated by quantifying the copy numbers of viral RNA. Figure 2B shows that there was no difference between the individual Arf-siRNA knockdown samples and the control siRNA treated samples, suggesting that single Arf depletion had no effect on viral replication.

### Double knockdowns of Arf1 and Arf3 inhibit EV71 replication in cells

**Figure 3:**
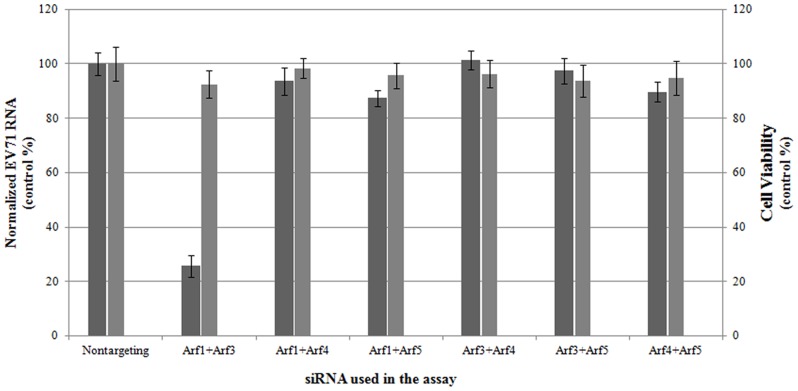
Double knockdown of Arf1 and Arf3 inhibits EV71 replication in RD cells. Of all the possible Arf pairs (omitting Arf2), only the Arf1+Arf3 double knockdown inhibited EV71 replication (*P*<0.05).

Different Arf isoforms generally act in pairs at distinct sites in the secretory pathway [28]. Therefore, we tested whether combined knockdowns of two Arf proteins would affect EV71 replication in cells. In Figure 3, double knockdowns of Arf1 and Arf3 (Arf1+3) reduced replication of EV71. Conversely, viral replication was not affected by any of the other double knockdowns tested in this study at 6 hpi, including Arf1+4, Arf1+5, Arf3+4, Arf3+5, and Arf4+5. In the latter groups, the levels of viral replication were similar to that observed in the control siRNA-treated group. Cell viability was also assessed by MTS assay to show that the inhibition of viral replication in cells with knockdown expression of Arf1+3 was not due to the toxicity of the siRNA treatment. In Figure 3, there was no significant difference for the cell viability between gene-specific siRNA treated groups and non-targeting siRNA treated controls. This result suggested that the reduction of viral replication was caused by double Arf1+3 knockdown.

### Overexpression of Arf proteins does not rescue viral replication from BFA exposure

**Figure 4:**
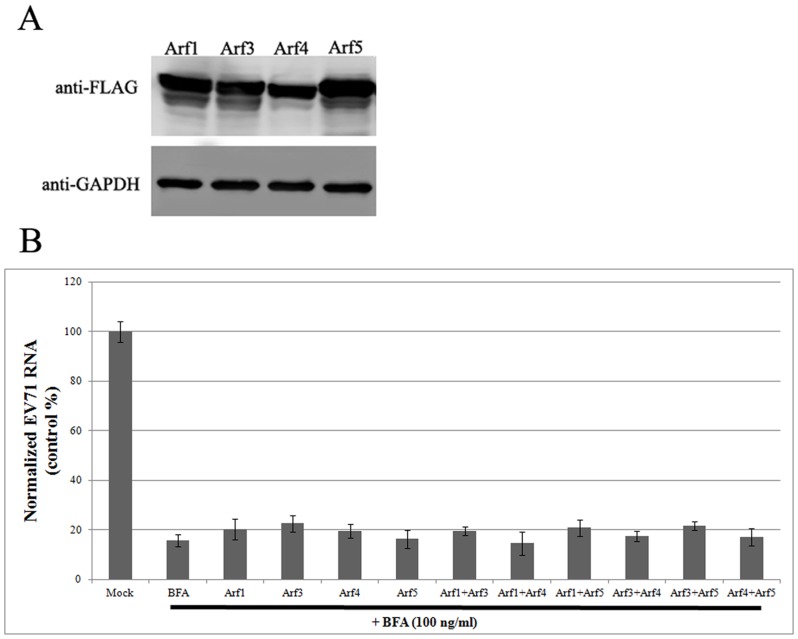
Overexpression of Arf proteins could not rescue EV71 replication from BFA exposure. (A) All the Arf proteins tested are expressed at high levels in RD cells. (B) Overexpression of Arf proteins did not rescue viral replication under BFA exposure (*P*>0.05).

To test the effect of Arf protein overexpression on EV71 replication, Arf1 and Arfs 3–5 were overexpressed in RD cells for 24 h. Figure 4A shows that all the Arf proteins were expressed at high levels in RD cells. EV71 virions were then used to infect (1 MOI) RD cells that overexpressed a single or a pair of Arf proteins. Control cells transfected with the empty vector were also infected. At the time of infection, BFA (100 ng/ml) was added to cell cultures. Total cellular RNA and viral RNA were extracted after 6 hpi. The copy numbers of EV71 RNA were detected by quantitative real-time PCR. Figure 4B shows that BFA substantially inhibited EV71 replication in RD cells. However, the overexpression of a single Arf was unable to rescue viral replication from BFA after 6 hpi. Surprisingly, the simultaneous overexpression of both Arf1 and Arf3 was also unable to rescue viral replication against BFA inhibition, not to mention overexpression of the other Arf combinations. Replication of EV71 RNA in both the Arf overexpressing groups and the control group was noticeably inhibited by BFA.

### GBF1 is required for EV71 replication

**Figure 5:**
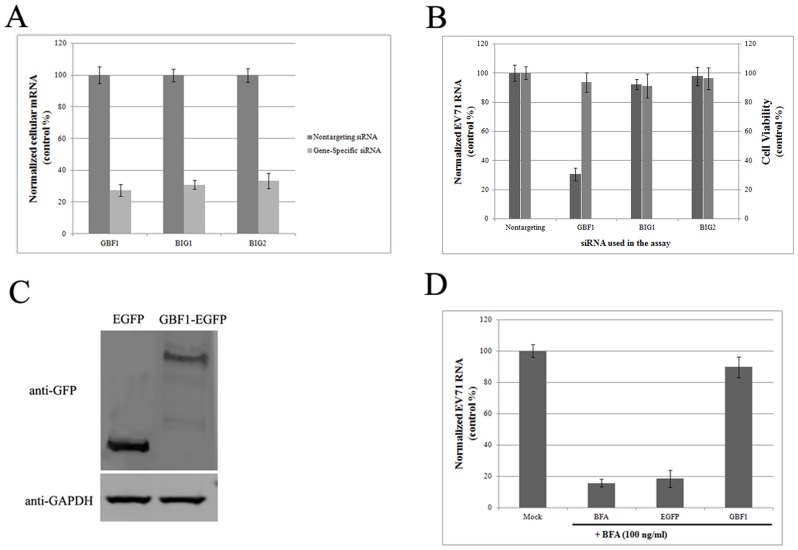
GBF1 is required for EV71 replication. (A) Targeted siRNAs could effectively knockdown the expression of each GEF (*P*<0.05). (B) Knockdown of GBF1, but not BIG1 or BIG2, inhibited EV71 replication in cells (*P*<0.05). (C) GBF1-EGFP is overexpressed in RD cells by transfection. (D) Overexpression of GBF1 rescued viral replication from BFA exposure (*P*<0.05).

To determine the contribution of the individual GEFs to EV71 replication, we also utilized siRNAs to individually knockdown BIG1, BIG2, and GBF1. The efficiency of siRNA treatment was first assessed by quantitative detection of the individual GEF mRNA transcripts. Figure 5A shows that the transcripts for each GEF were reduced by approximately 70% with specific siRNA treatments. EV71 (1 MOI) was then used to infect GEF knockdown cells. At 6 hpi, total cellular RNA was extracted, and quantitative detection of the viral RNA genome was performed. Figure 5B shows that the BIG1 and BIG2 knockdowns did not alter viral replication compared to the control siRNA-treated group. However, when GBF1 was depleted with siRNA, EV71 replication was substantially inhibited. Cell viability was also tested. No noticeable difference was detected between GBF1 knockdown group and control cells, indicating that the reduction of viral replication was not due to cell toxicity of siRNA treatment (Figure 5B). The essential role for GBF1 in EV71 replication was further checked with BFA treatment. RD cells were transfected with a plasmid that expressed GBF1 fused to the enhanced green fluorescent protein (GBF1-EGFP). The pEGFP-N1vector was also transfected into cells as a control. Figure 5C shows that GBF1-EGFP was overexpressed in RD cells. The ability of GBF1 to rescue EV71 replication from BFA exposure was then tested. BFA (100 ng/ml) substantially inhibited EV71 replication in control cells (Figure 5D). However, the overexpression of GBF1-EGFP, but not EGFP alone, rescued viral replication from BFA exposure (Figure 5D).

### GBF1 interacts with viral 3A protein

**Figure 6:**
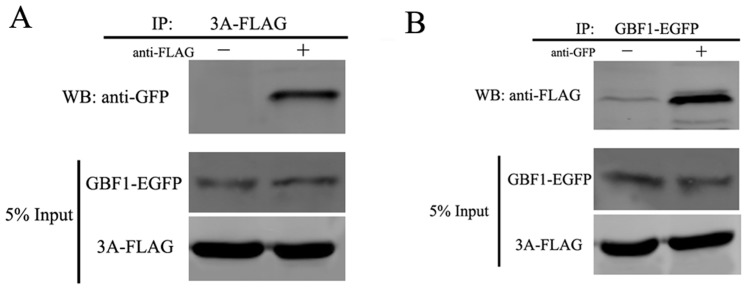
GBF1 interacts with viral 3A protein. (A) Immunoprecipitation was conducted with Protein G agarose plus anti-FLAG antibody. Western blots were probed with specific antibodies, as indicated. (B) Immunoprecipitation was conducted with Protein G agarose plus anti-GFP antibody. Western blots were probed with specific antibodies, as indicated.

GBF1-EGFP was overexpressed in 293T cells that were cotransfected with the EV71 3A protein fused to the FLAG tag (3A-FLAG). A direct interaction between GBF1 and the EV71 3A protein was confirmed by immunoprecipitation with the FLAG-tag, followed by immunoblotting and probing for the GBF1-EGFP protein. Figure 6A shows that GBF1-EGFP was captured in immunoprecipitations of the 3A-FLAG protein. The reverse immunoprecipitation of GBF1-EGFP with the GFP antibody was also conducted, followed by probing the immunoblots for 3A-FLAG. The 3A-FLAG protein was captured in immunoprecipitations of the GBF1-EGFP protein (Figure 6B). Taken together, these results suggested that GBF1 interacted directly with the viral 3A protein.

## Discussion

Picornaviruses induce the formation of a cytoplasmic vesicular compartment in infected cells, and this compartment is the essential site of viral RNA replication. It has been well documented that picornavirus replication was associated with membranes derived from the endoplasmic reticulum via a COPII coatamer-mediated process or from the Golgi via a COPI-mediated process [7], [29], [30]. Our previous findings also revealed an essential role of cellular COPI activity in EV71 replication [11]. A component of the COPI coatamer, β-COP, is recruited to membranes. This recruitment is regulated by the small cellular GTPase, Arf1 [30]. Taken together, those findings suggested an Arf1-dependent membrane trafficking step may be required for EV71 replication. In the present report, we characterized the role of Arfs in EV71 replication. We demonstrated that EV71 replication required both Arf1 and Arf3 combined, and the large GEF, GBF1.

Five out of six Arfs are expressed in human cells. Arfs 1, 3, 4, and 5 are functionally involved in intracellular membrane trafficking. Once activated, the membrane-associated Arf-GTP induces a curvature in the lipid bilayer, which in turn, facilitates the formation of secretory vesicles. Membrane-bound Arf1 can also recruit a diverse array of effectors, including COPI, clathrin, cytoskeletal regulators, and lipid-modifying enzymes [30], [31]. Arf1 has been shown to colocalize with the enteroviral replication machinery [18], [32]. Poliovirus 3A and 3CD protein synthesis was found to induce the translocation of Arf1 to membranes [33], [34]. The substitution of the F441 residue to S441 in the viral 3CD protein caused a loss of function in Arf1 translocation activity, and it was lethal for the virus [33]. In addition, cell-free poliovirus replication was inhibited by adding peptides from the N-terminus of Arf1; this result suggested that Arf1 activity played a significant role in viral replication [35]. Arf1, Arf3, and Arf5 were reported to be activated upon poliovirus infection [33], [34]. Our results also demonstrated that Arf1 and Arf3 were upregulated and activated in EV71 infections. However, our knockdown results showed that a single knockdown of either Arf1 or any other Arf had no effect on EV71 replication. This was consistent with Lanke's result that Arf1 was dispensable for coxsackievirus B3 replication [26]. This might be explained by the high homology of the Arf proteins, which may enable Arfs to compensate for one another to preserve cellular functions. In the Class I Arfs, Arf1 and Arf3 are 96% identical; the Class II Arfs, Arf4 and Arf5, are 90% identical; the Class I and II Arfs exhibit about 80% identity, and Arf6 is only about 70% identical to the other Arfs. When we tested combinations of double Arf knockdowns, we found that knocking down both Arf1 and Arf3 inhibited EV71 replication in cells. Volpicelli-Daley *et al* showed that Arf1 and Arf3 were important for protein transport between the endoplasmic reticulum and the Golgi network, and double knockdowns of Arf1 and Arf3 led to a redistribution of COPI proteins from the Golgi membranes to the cytosol [28]. This was consistent with our previous findings that COPI activity was required for EV71 replication [11]. In contrast, other combined double knockdowns did not affect viral replication.

Although many studies point to the involvement of different Arf proteins in picornavirus replication, our observations, together with other reports, showed that Arf overexpression could not rescue virus replication from BFA exposure. This might be explained by the fact that picornavirus replication requires activated Arf-GTP, which is controlled by GEFs. Mammalian cells contain three large Arf –associated GEFs, GBF1, BIG1, and BIG2, and all are sensitive to BFA [36]. Indeed, it was thought that all three of these GEFs were likely to be involved in enterovirus replication. Several studies showed that Arf1 translocation, induced by the enteroviral 3A protein, was dependent on cellular GBF1 activity; in contrast, Arf1 activation, induced by the enteroviral 3CD protein, was dependent on BIG1 and BIG2 activities [18], [34], [37]. Other reports demonstrated a direct interaction between cellular GBF1 and the viral 3A protein of either poliovirus or coxsackievirus B3 [37], [38], [39]. In the present study, we demonstrated a crucial role for GBF1 in EV71 replication. We found that the siRNA-mediated depletion of GBF1, but not BIG1 or BIG2, inhibited EV71 replication. Additionally, GBF1 overexpression rescued EV71 replication in the presence of BFA. Our co-immunoprecipitation results also showed that GBF1 directly interacted with the EV71 3A protein. Although the involvement of GBF1 in replication may be universal for enterovirus, the exact role of GBF1 in the formation of the viral replication complex requires further study.

There are several possible roles that GBF1 could play in viral replication. For example, GBF1 binding to the viral 3A protein may recruit phosphatidylinositol 4-phosphate (PI4P) to the viral replication complex. PI4P is the principle phosphoinositide in the Golgi apparatus, and it acts as a targeting signal for Golgi-associated proteins [40]. EV71 replication requires the activity of PI4P [41], and Arf1 and GBF1 were shown to generate a PI4P-enriched environment to support viral replication [20]. Another role for GBF1 might be that the virus sequesters GBF1 to disrupt the secretory pathway in infected cells; in turn, this disruption could block the secretion of interferons and proinflammatory cytokines, or it could inhibit cell surface MHC class 1 expression and antigen presentation. Indeed, it has been widely observed that the enteroviral 3A protein alone could inhibit cellular secretory pathway transport [38], [42], [43]. The finding that the viral 3A protein also interacted with cellular GBF1 might suggest that the viral 3A protein and EV71 act through a similar pathway. The up-regulation of Arf1 and 3 expression may also represent a cellular compensatory response for disregulation of the GBF1-dependent pathway in EV71-infected cells. The failure to rescue the replication from BFA by overexpression of any Arf combinations was a strong argument against their real requirement for replication. It was also previously shown for coxsackievirus B3 that overexpression of the activated mutants of Arf 1 and 3 either separately or in combination also cannot rescue replication form BFA block [26]. Moreover, mutations that fail to inhibit protein secretion are not lethal to enterovirus replication [44]. Thus the inhibition of protein transport may not be absolutely required for replication in cultured cells, but play a role in the virus's evasion of the host's immune responses.

In conclusion, this study was the first to demonstrate that Class I Arfs were involved in EV71 replication and that the combined double knockdown of Arf1 and Arf3 inhibited viral replication. Additionally, we confirmed that GBF1 played a crucial role in EV71 replication.

## Materials and Methods

### Cell culture and drug treatment

RD cells (ATCC, USA) and HEK293T (293T) cells (ATCC, USA) were propagated and maintained in Minimum Essential Medium (MEM; HyClone, Logan, USA) and Dulbecco's Modified Eagle Medium (DMEM; HyClone, Logan, USA), supplemented with 10% fetal bovine serum (FBS; Invitrogen, CA, USA), 100 U/ml penicillin, and 100 µg/ml streptomycin, at 37°C with 5% CO_2_. BFA (Sigma-Aldrich, St. Louis, USA) was dissolved in ethanol and stored at 1 mg/ml at 4°C before use.

### Virus infection

The EV71 strain Shzh-98 (GenBank accession no. AF302996) was used in this study. Viruses were propagated in RD cells and infected at a MOI of one per cell, based on the 50% tissue culture infectious dose (TCID_50_).

### Plasmid construction

For transient expression in 293T cells, the EV71 3A sequence was cloned from the EV71 Shzh-98 strain and inserted into a pcDNA3 vector (Invitrogen, CA, USA) under the control of the cytomegalovirus (CMV) promoter. A FLAG-tag sequence (amino acids: DYKDDDDK) was inserted at the 3′ end of the 3A coding region to produce a 3A-FLAG-tagged protein, which could facilitate co-immunoprecipitation and Western blot detection. The human *GBF1* gene was cloned into the pEGFP-N1 vector (Clontech, Heidelberg, Germany) and transfected into 293T cells for transient expression. The nucleotide sequences of the plasmids and the orientation of the inserted fragments were verified by DNA sequencing.

### siRNA design and transfection

To ensure efficient knockdown, we used a mixture two specific siRNAs that targeted two different sequences on each gene. The siRNAs used in the knockdown assay are listed in Table 1 [28]. The stealth siRNA negative control Med GC (Invitrogen, CA, USA) was used as a negative control. siRNA was introduced into RD cells by transfection with Oligofectamine Reagent (Invitrogen, CA, USA), according to the manufacturer's instructions. RD cells were cultured overnight, and they were transfected at 40% confluence. Before transfection, 100 pmol siRNA was incubated in 60 µl Opti-MEM. (Invitrogen, CA, USA) for 15 min at room temperature (RT) and 5 µl Oligofectamine was incubated in 15 µl Opti-MEM. The siRNA and Oligofectamine were then mixed and incubated for 20 min at RT before adding to cell cultures. At 4 h after transfection, the culture medium was changed, and cells were cultured for 72 h before the virus infection.

**Table 1:** siRNA sequences used in this study.

Target Gene	Duplex	Sense Sequence
Arf1	1	5′-ACCGTGGAGTACAAGAACATT-3′
	2	5′-TGACAGAGAGCGTGTGAACTT-3′
Arf3	1	5′-TGTGGAGACAGTGGAGTATT-3′
	2	5′-ACAGGATCTGCCTAATGCTT-3′
Arf4	1	5′-TCTGGTAGATGAATTGAGATT-3′
	2	5′-AGATAGCAACGATCGTGAATT-3′
Arf5	1	5′-TCTGCTGATGAACTCCAGATT-3′
	2	5′-CCATAGGCTTCAATGTAGATT-3′
GBF1	1	5′-CCACCAGAACATGGGAAATT-3′
	2	5′-GCTCTCAGCAGTGAGTCTATT-3′
BIG1	1	5′-GCACCACACCTGAAGATATT-3′
	2	5′-GCAGCTTTCCCAGACACAATT-3′
BIG2	1	5′-GCACATCCCTTGACTGCTT-3′
	2	5′-GCGGCTGCTGTACAACTTATT-3′

### Quantitative real-time PCR

Total cellular RNA and viral RNA were extracted from each cell culture well with the RNAeasy Mini kit (Qiagen, Hilden, Germany) at 6 hpi, according to the manufacturer's instructions. Reverse transcription and PCR were conducted with the Superscript First-Strand Synthesis System (Invitrogen, CA, USA) in a volume of 20 µl, with 1.2 µg total RNA, according to the manufacturer's protocol. Relative quantitative real-time PCR was conducted on an ABI Prism 7000 Real-time PCR System (Applied Biosystems, CA, USA). The PCR reactions contained 2 µl cDNA, 1 µl of each primer, and 25 µl Power SYBR Green PCR Master Mix (Applied Biosystems, CA, USA) in a total volume of 50 µl. Efficiency-corrected, relative quantification was performed, with GAPDH as an internal control [45]. Primer sequences are shown in Table 2.

**Table 2:** Quantitative real-time PCR primers.

Target	Forward Primer	Reverse Primer
EV71	CCCCTGAATGCGGCTAAT	CAATTGTCACCATAAGCAGCCA
GAPDH	CTCTGCTCCTCCTGTTCGAC	TTAAAAGCAGCCCTGGTGAC
Arf1	TGCGGCCAGGCTTTTTATTTA	TGCTGCGCCACCACCACCTCAT
Arf3	GATTGGGAAGAAGGAGATGC	GTTACGGTGACGAAGGGAATG
Arf4	GGGGAGATAGTCACCACCATT	GCCAGTCAAGTCCTTCATACAG
Arf5	GGGGAGATTGTCACCACCAT	CAGCCAGTCCAGACCATCGTA
GBF1	GGAAGAGACACCATCAAACC	CAGGCGTAGCAACCCCACCAC
BIG1	GTTGGCTCTGTGCTGTGTCTA	ATGTGCCCGGTTGTTTGGAATG
BIG2	AGAGGCCTCGGGTGCTAC	ATCGCGCCAACTGTTCATTATC

### Western blotting

RD cells were collected and washed with phosphate-buffered saline (PBS) twice, then lysed in lysis buffer containing 100 mM NaCl, 20 mM Tris (pH 8.0), 0.5% NP-40, 0.25% sodium deoxycholate, 1 mM EDTA, and proteinase inhibitor cocktail. The lysate was centrifuged at 15,000×*g* for 15 min, and the supernatant was collected. Proteins were then separated by electrophoresis in a denaturing, 4 to 10% polyacrylamide gel (SDS-PAGE). The separated proteins were transferred to nylon polyvinylidene difluoride (PVDF) membranes (Hybond P, Piscataway, USA). The membranes were then blocked with 5% nonfat dry milk, and probed with primary antibodies, as indicated, at 4°C overnight. The probes were visualized by incubation with the corresponding IRD Fluor 680-labeled IgG secondary antibody (Li-Cor Inc., NE, USA). After washing, membranes were scanned with an Odyssey Infrared Imaging System (Li-Cor, NE, USA) at the recommended wavelength and analyzed with Odyssey software. Molecular sizes of proteins were determined by comparison with prestained protein markers (Fermentas, Maryland, USA). Human Arf1 and Arf3 were detected with anti-Arf1+3 polyclonal antibodies (Abcam, Cambridge, England). EGFP and the EGFP-fusion GBF1 protein were identified with mouse anti-GFP (Beyotime, Suzhou, China). The 3A-FLAG protein was detected with mouse anti-FLAG antibody (Beyotime, Suzhou, China) and a corresponding secondary antibody. To control for protein loading, expression of the housekeeping protein, GAPDH and calnexine were assessed with mouse anti-GAPDH (Beyotime, Suzhou, China), rabbit anti-calnexine (Abcam, Cambridge, England) and IRD Fluor 680-labeled IgG secondary antibody (Li-Cor Inc., NE, USA).

### Transfection and co-immunoprecipitation assay

Cell transfection was performed with Lipofectamine 2000 (Invitrogen, CA, USA) according to the manufacturer's protocol. Briefly, 10 µg of plasmids were mixed with 30 µl Lipofectamine 2000 and incubated at RT for 20 min before adding to the cell cultures. Transfected 293T cells were cultured for another 24 h. Cells were collected and lysed in lysis buffer. The lysates were centrifuged at 15,000×*g* to remove debris, and the supernatants were incubated with Protein G agarose (Invitrogen, CA, USA) and 2 µg mouse anti-FLAG antibody (Beyotime, Suzhou, China) overnight at 4°C. After a brief centrifugation, the immunocomplexes were washed three times with PBS and subjected to SDS-PAGE. Protein bands were detected by Western blotting with the anti-GFP antibody. A reciprocal co-immunoprecipitation assay was conducted by adding Protein G agarose (Invitrogen, CA, USA) and 2 µg mouse anti-GFP antibody (Beyotime, Suzhou, China) to cell protein extracts overnight at 4°C. After a brief centrifugation, the immunocomplexes were washed three times in PBS and subjected to SDS-PAGE. The proteins were transferred to Western blots, and probed with the anti-FLAG antibody.
